# Radiation Dose Reduction in Mechanical Thrombectomy: Single Versus Dual-Operator Approach

**DOI:** 10.3390/tomography12020014

**Published:** 2026-01-23

**Authors:** Mustafa Demir, Yunus Yasar

**Affiliations:** Department of Radiology, Umraniye Training and Research Hospital, University of Health Sciences, Kazım Karabekir Mah, Adem Yavuz Cd F No:10, Istanbul 34899, Turkey; dryunusysr@gmail.com

**Keywords:** mechanical thrombectomy, radiation exposure, work efficiency, dual-operator, clinical outcomes, neuroradiology

## Abstract

**Objective:** The number of operators performing mechanical thrombectomy (MT) may influence procedural outcomes; however, evidence remains limited and conflicting. This study aimed to comprehensively evaluate the impact of single versus dual operators on procedure time, radiation dose, and angiographic success in patients undergoing MT for acute ischemic stroke. **Methods:** In this single-center, retrospective cohort study, 285 consecutive patients who underwent MT for large-vessel occlusion between January 2020 and December 2024 were included. Patients were grouped according to institutional workflow: single-operator procedures (*n* = 157) and dual-operator procedures (*n* = 128). The primary endpoints were procedure time and radiation dose parameters, including total Kerma-Area Product (PKA). Secondary endpoints included successful reperfusion (TICI ≥ 2b), complete reperfusion (TICI 3), and first-pass success (FPS, defined as TICI 2c/3 with a single pass). **Results:** Baseline characteristics were comparable between groups. The dual-operator group had significantly shorter median procedure times (52.5 vs. 85.0 min, *p* < 0.001) and lower total PKA (*p* < 0.001). Reperfusion rates were significantly higher in the dual-operator group, both for successful reperfusion (TICI ≥ 2b: 80.5% vs. 64.3%, *p* = 0.004) and complete reperfusion (TICI 3: 76.6% vs. 58.5%, *p* = 0.002). First-pass success was also more frequent (60.0% vs. 44.5%, *p* = 0.0146), and the mean number of passes was lower (1.66 vs. 2.00, *p* = 0.0057). **Conclusions:** Mechanical thrombectomy performed with two experienced operators was associated with greater procedural efficiency, reduced patient radiation exposure, and higher angiographic success compared with single-operator procedures. These findings support considering the dual-operator model as an approach that may inform workforce planning and workflow decisions in stroke centers.

## Simple Summary

Mechanical thrombectomy is an effective treatment for acute ischemic stroke but involves radiation exposure during imaging. In clinical practice, procedures may be performed by one or two experienced radiologists. In this study, we compared single- and dual-operator thrombectomy procedures. We found that procedures performed by two operators were associated with shorter procedure times, lower radiation exposure, and higher rates of successful vessel reopening. These findings suggest that a dual-operator approach may improve procedural efficiency and patient safety, and support future studies aimed at optimizing thrombectomy workflows.

## 1. Introduction

Acute ischemic stroke is one of the leading causes of long-term disability and mortality in the adult population worldwide. A significant proportion of these cases result from large-vessel occlusions (LVOs), for which the efficacy of intravenous thrombolysis remains limited [1]. Over the past decade, several randomized controlled trials have conclusively demonstrated that mechanical thrombectomy (MT) provides substantial functional and clinical benefits compared to standard medical therapy in patients with LVO, thereby establishing MT as the standard of care [1,2,3,4,5]. The success of MT largely depends on achieving rapid and complete reperfusion. It is well established that reperfusion graded as Thrombolysis in Cerebral Infarction (TICI) 2b or higher is associated with improved clinical outcomes [1,2]. More recent evidence further suggests that near-complete or complete reperfusion (TICI 2c/3) correlates with even better long-term functional outcomes [6,7].

MT procedures are inherently performed under fluoroscopic guidance, which inevitably exposes both patients and operators to ionizing radiation. Radiation exposure carries not only deterministic risks, such as skin erythema and epilation, but also stochastic risks, including an increased lifetime risk of malignancy. Therefore, minimizing radiation exposure according to the “As Low As Reasonably Achievable (ALARA)” principle is of critical importance [8]. In line with this, diagnostic reference levels (DRLs) for interventional neuroradiology have been established and are regularly updated in many countries [8,9,10].

Multiple factors have been shown to influence procedure time and radiation dose. The introduction of new-generation angiography systems and radiation dose-reduction software (RDS) has markedly reduced exposure for both patients and operators without compromising image quality [10,11]. Similarly, the use of low-dose fluoroscopy and digital subtraction angiography (DSA) protocols has been shown to effectively reduce radiation burden [12]. The choice of thrombectomy technique is also important; direct aspiration (ADAPT) has been reported to shorten procedure time and decrease radiation exposure compared to stent retrievers [13]. The complexity of the intervention, particularly in cases with tandem occlusions or when additional intracranial/extracranial stenting is required, is another determinant of both procedure time and dose [14]. Among the most influential factors, however, is the number of recanalization attempts required, since each additional pass significantly increases radiation exposure [3,8,9]. Operator experience represents another critical determinant: it has been demonstrated that experienced operators, particularly after surpassing the 25–50 case learning curve, achieve shorter procedure and fluoroscopy times with lower radiation doses [15,16].

Another potential factor influencing procedural efficiency, though relatively underexplored in the literature, is the number of operators performing the thrombectomy. In clinical practice, especially in low-volume centers or complex cases, procedures are often conducted by two operators. Theoretically, a dual-operator approach may facilitate catheter manipulation, reduce procedure and fluoroscopy times, decrease radiation exposure, and ultimately improve reperfusion outcomes. However, existing studies are scarce, and their results remain inconsistent [17].

The aim of this study was to comprehensively evaluate the effect of single- versus dual-operator approaches on procedure time, radiation dose, and both overall and first-pass reperfusion success in patients undergoing MT for acute ischemic stroke. Our hypothesis was that procedures performed with dual operators would be associated with shorter procedure times, lower patient radiation exposure, and higher reperfusion success rates.

## 2. Materials and Methods

### 2.1. Study Design and Patient Population

This retrospective single-center cohort study included all consecutive patients aged ≥18 years who underwent mechanical thrombectomy (MT) for acute ischemic stroke due to intracranial large vessel occlusion (LVO) between January 2020 and December 2024. The study was approved by the institutional ethics committee, and the requirement for individual informed consent was waived because of the retrospective design. This study is reported in accordance with the Strengthening the Reporting of Observational Studies in Epidemiology (STROBE) statement.

Eligible occlusion sites included the terminal internal carotid artery (ICA), tandem occlusions, the middle cerebral artery (MCA) M1 and M2 segments, the anterior cerebral artery (ACA) A1 and A2 segments, and posterior circulation occlusions involving the basilar artery and posterior cerebral artery (PCA).

### 2.2. Study Groups

Patients were categorized according to the number of interventional neuroradiologists who performed the procedure:

- Single-operator group: procedures performed entirely by a single board-certified interventional neuroradiologist with >5 years of independent experience, from arterial puncture to final angiography, without active technical assistance from a second operator.
- Dual-operator group: procedures jointly performed by two board-certified interventional neuroradiologists, each with >5 years of independent experience. In these cases, one operator acted as the primary operator, while the second provided active assistance in catheter navigation and device manipulation, as well as procedural decision-making support.

In our institution, this grouping reflected institutional workflow rather than case complexity. During regular working hours, all thrombectomy procedures were routinely performed by two experienced operators, whereas during on-call hours, procedures were performed by a single operator, as a second interventional neuroradiologist was not routinely available. Thus, the assignment to single- or dual-operator groups was determined by organizational factors rather than clinical or anatomical selection.

### 2.3. Angiographic System and Procedural Details

All procedures were performed under general anesthesia using a monoplane angiographic system (Artis zee, Siemens Healthineers, Erlangen, Germany). A standard transfemoral approach was used in all cases.

According to institutional protocol, an appropriately sized guiding catheter was positioned in the cervical ICA. The choice of first-line thrombectomy technique—direct aspiration, stent retriever, or the combined Solumbra technique—was left to the operator’s discretion, taking into account clot characteristics and vascular anatomy. Final reperfusion status was assessed on completion angiography using the Thrombolysis in Cerebral Infarction (TICI) grading scale.

### 2.4. Data Collection

Clinical, demographic, and imaging data were retrospectively collected from the hospital information system and the picture archiving and communication system (PACS). Extracted variables included:

- Demographic and clinical data: age, sex, baseline National Institutes of Health Stroke Scale (NIHSS) score, Alberta Stroke Program Early CT Score (ASPECTS), and intravenous tissue plasminogen activator (tPA) administration.
- Angiographic data: Occlusion site.
- Procedural data: first-line thrombectomy technique, procedure time (minutes), fluoroscopy time (seconds), digital subtraction angiography (DSA) run time (seconds), and number of passes.
- Radiation dose data: total kerma-area product (PKA, Gy·m^2^), fluoroscopy PKA, and imaging (DSA) PKA, all automatically recorded by the angiographic system. These parameters represent patient radiation exposure surrogates widely used in interventional radiology studies; direct dosimeter-based operator dose measurements were not performed, as the present study focused on patient exposure rather than occupational radiation dose.
- Angiographic outcomes: success of selective catheterization, reperfusion success defined as TICI ≥ 2b, complete reperfusion defined as TICI 3, first-pass success (FPS), and modified first-pass effect (mFPE).

### 2.5. Outcomes and Definitions

The primary endpoints were total procedure time and radiation dose parameters (total PKA, fluoroscopy PKA, and imaging PKA).

Procedure time was defined as the interval from femoral artery puncture to the final angiographic run documenting the last reperfusion result. Digital subtraction angiography (DSA) run time was defined as the cumulative duration of all DSA acquisitions performed during the thrombectomy procedure, excluding fluoroscopy-only periods.

Selective catheterization success was defined as the successful selective catheterization of the target intracranial vessel supplying the occluded segment, allowing delivery of the thrombectomy device, irrespective of the specific vascular level. This metric primarily reflected access-related challenges, including unfavorable aortic arch anatomy or supra-aortic vessel configuration, rather than intracranial navigation beyond the cervical internal carotid artery.

The secondary endpoints were angiographic measures of procedural effectiveness. Successful reperfusion was defined as TICI ≥ 2b, while complete reperfusion was defined as TICI 3. First-pass success (FPS) was defined as achieving near-complete or complete reperfusion (TICI ≥ 2c) after a single thrombectomy pass. Early reperfusion without rescue (ERR) was defined as achieving near-complete or complete reperfusion (TICI ≥2c) within up to three thrombectomy passes without the use of any rescue therapy, representing an operational measure of early procedural effectiveness without escalation to additional techniques.

### 2.6. Statistical Analysis

All statistical analyses were performed using SPSS version 25.0 (IBM, Armonk, NY, USA). Continuous variables were tested for normality. Normally distributed variables were compared using Student’s *t*-test, and non-normally distributed variables using the Mann–Whitney U test. Categorical variables were analyzed using the Chi-square or Fisher’s exact test as appropriate. Statistical significance was set at *p* < 0.05.

## 3. Results

A total of 285 patients were included in the study; 157 (55.1%) were treated by a single operator and 128 (44.9%) by dual operators. The median age was 75 years (IQR, 62–82), and 51.6% of the cohort were male. Baseline clinical characteristics, including age, sex, admission NIHSS score, ASPECTS, and rate of intravenous tPA administration, showed no significant differences between groups (all *p* > 0.05). The distribution of occlusion sites was as follows: 24.2% ICA terminal/tandem, 43.5% MCA M1–ACA A1, 18.6% MCA M2–ACA A2, and 13.7% basilar/PCA, without significant intergroup differences (Table 1).

The distribution of first-line thrombectomy techniques was comparable between groups (*p* = 0.289). Direct aspiration was used in 41.4% of cases, stent retriever in 16.1%, and Solumbra in 42.5% (Table 2).

Median procedure time was significantly shorter in the dual-operator group (52.5 min [IQR, 30.0–70.0]) compared with the single-operator group (85.0 min [IQR, 60.0–120.0], *p* < 0.001). Dual-operator procedures were also associated with lower fluoroscopy time (1114.0 s [IQR, 718.5–2018.0] vs. 1627.0 s [IQR, 1040.0–2335.0], *p* < 0.001) and shorter DSA run time (52.0 s [IQR, 36.0–78.2] vs. 64.0 s [IQR, 41.0–103.0], *p* = 0.026). In terms of patient radiation exposure, total kerma–area product (PKA) was significantly lower in the dual-operator group (0.0063 Gy·m^2^ [IQR, 0.0041–0.0100]) compared with the single-operator group (0.0092 Gy·m^2^ [IQR, 0.0060–0.0130], *p* < 0.001). Similarly, both fluoroscopy PKA (0.0023 vs. 0.0035 Gy·m^2^, *p* < 0.001) and imaging PKA (0.0038 vs. 0.0050 Gy·m^2^, *p* = 0.031) were lower in the dual-operator group (Table 3).

When stratified by occlusion site, procedure times were significantly shorter in the dual-operator group for ICA terminal/tandem (*p* = 0.0063), MCA M1/ACA A1 (*p* < 0.001), and basilar/PCA occlusions (*p* = 0.0016). Fluoroscopy time was significantly lower in MCA M1/ACA A1 occlusions (937.0 s vs. 1632.0 s, *p* = 0.0011). Total PKA was significantly reduced in the dual-operator group for MCA M1/ACA A1 occlusions (0.0058 vs. 0.0097 Gy·m^2^, *p* < 0.001) and MCA M2/ACA A2 occlusions (0.0058 vs. 0.0093 Gy·m^2^, *p* = 0.0046), while no significant difference in total PKA was observed for ICA terminal/tandem or basilar/PCA occlusions (Table 4).

Selective catheterization success rates were high in both groups (94.3% vs. 97.7%, *p* = 0.24). Among patients with successful selective catheterization, dual operators achieved higher reperfusion rates than single operators for both TICI ≥ 2b (80.5% vs. 64.3%, *p* = 0.004) and TICI 3 (76.6% vs. 58.5%, *p* = 0.002) (Table 5).

Subgroup analysis by occlusion level revealed that in ICA terminal/tandem occlusions, dual operators achieved significantly higher rates of both TICI ≥ 2b (96.0% vs. 77.3%, *p* = 0.0472) and TICI 3 reperfusion (88.0% vs. 56.8%, *p* = 0.0080). In MCA M1/ACA A1 occlusions, a trend toward higher TICI 3 rates was observed in the dual-operator group (77.8% vs. 60.7%, *p* = 0.0513) (Table 6).

First-pass success (FPS) was significantly higher in the dual-operator group (60.0% vs. 44.5%, *p* = 0.0146). Early reperfusion without rescue (ERR) rates were similarly high in both groups (92.0% vs. 88.4%, *p* = 0.4164). The mean number of passes was significantly lower with dual operators (1.66 ± 1.02) compared with single operators (2.00 ± 1.34, *p* = 0.0057) (Table 7).

## 4. Discussion

### 4.1. Main Findings

This study demonstrated that performing mechanical thrombectomy (MT) for acute ischemic stroke with two experienced operators was associated with significantly shorter procedure times, lower patient radiation exposure, as reflected by reduced kerma–area product (PKA) values, and higher angiographic success rates than with a single operator. These findings indicate an association between a dual-operator approach and improved procedural efficiency and angiographic outcomes, while the observational design precludes causal inference regarding the independent effect of operator number.

### 4.2. Comparison with Literature

The median procedure time in the single-operator group (85.0 min) was comparable to times reported in studies in which stent retriever-based strategies predominated [18]. In contrast, the dual-operator group achieved substantially shorter procedure times (52.5 min), approaching those reported for direct aspiration techniques, which are generally considered faster [13]. This approximately 38% reduction in procedure time represents a marked difference between groups and serves as a key indicator of procedural efficiency associated with the dual-operator approach.

Radiation exposure in the single-operator group was consistent with values reported in large international registries and in centers utilizing modern dose-reduction systems (RDS) [8,9,10]. The significant dose reduction observed in the dual-operator group is consistent with improved adherence to ALARA principles and is likely related to shorter procedure duration and fewer thrombectomy passes. The primary mechanism for reduced patient radiation exposure is likely the decrease in procedure duration and, importantly, the lower number of passes required for successful reperfusion. Prior studies have shown that each additional pass significantly increases both total radiation exposure and complication risk [3,8,9]. Our results corroborate this mechanism, as the mean number of passes was significantly lower in the dual-operator group (1.66 vs. 2.00).

In addition to procedural and organizational factors, anesthetic management has been recognized as a potential modifier of mechanical thrombectomy workflow and outcomes, and the choice between general anesthesia and conscious sedation remains an area of ongoing debate in the literature. Although all procedures in the present study were performed under general anesthesia according to institutional protocol, anesthetic strategy should be considered when interpreting thrombectomy outcomes across different centers [19].

### 4.3. Mechanistic Explanation

Beyond procedural efficiency, dual-operator procedures also demonstrated superior angiographic outcomes. Both successful reperfusion (TICI ≥ 2b) and complete reperfusion (TICI 3) rates were significantly higher in the dual-operator group, comparable to the best results reported in randomized controlled trials and large registries [1,2,3,4,5,6]. Notably, first-pass success (FPS)—a key contemporary benchmark in MT practice—was also significantly higher in the dual-operator group (60.0% vs. 44.5%). First-pass success was therefore interpreted strictly as a procedural performance metric rather than a surrogate for clinical outcome in the present study.

The underlying mechanisms likely involve division of labor and parallel workflow. While one operator focuses on the navigation and manipulation of distal devices such as microcatheters, stent retrievers, or aspiration catheters, the second operator can maintain guide catheter stability, perform angiographic runs, assist with device preparation, and provide real-time strategic input. This synergy facilitates more efficient and effective completion of each maneuver, thereby reducing both overall procedure duration and the number of passes required. As highlighted by Pasarikovski et al., dual-operator practice is often used in low-volume centers during their initial phases or in particularly complex cases [17]. Our findings extend this concept by suggesting that a dual-operator model may be associated with favorable procedural metrics even in cases beyond highly complex ones, warranting further evaluation before broader routine adoption.

### 4.4. Limitations

An important limitation of this study concerns the group assignment’s organizational nature. Procedures performed by dual operators predominantly occurred during regular working hours, whereas single-operator procedures were more frequently performed during on-call periods. This distinction introduces potential organizational confounding related to staffing levels, operator fatigue, and overall workflow efficiency, factors that could not be directly measured or fully adjusted for in the present retrospective analysis.

Consequently, these organizational differences limit causal inference and raise the possibility of residual confounding. Although baseline clinical characteristics and first-line thrombectomy techniques were comparable between groups, the absence of multivariable or propensity-based adjustment precludes definitive attribution of observed differences solely to the number of operators.

Furthermore, procedures performed during on-call or off-hours may be subject to additional organizational constraints compared with those performed during regular working hours. Emergency stroke interventions during nights or weekends have been associated with longer treatment times and less favorable outcomes, a phenomenon commonly referred to as the “weekend effect.” This represents an additional potential source of bias in retrospective comparisons of procedural efficiency and angiographic outcomes between groups [20].

In addition, the dual-operator workflow evaluated in this study reflects an institutional organizational model and may not be universally applicable across different healthcare systems. In many regions, workforce limitations necessitate task-shifting or task-sharing strategies, in which complex endovascular procedures are performed by a single operator with varying levels of support. Therefore, the feasibility and generalizability of a routine dual-operator approach may be constrained by local staffing structures, training paradigms, and resource availability [21].

Additional limitations include the single-center, retrospective design, which may limit generalizability, and the absence of long-term functional outcomes, such as 90-day modified Rankin Scale scores. While all operators had more than five years of independent experience, unmeasured individual performance differences may also have influenced procedural metrics.

### 4.5. Clinical Implications and Future Directions

Taken together, our results suggest that performing MT with two experienced operators is associated with greater procedural efficiency, lower patient radiation exposure, as reflected by reduced kerma-area-product (PKA) values, and higher rates of angiographic success. These findings may help inform workforce planning and workflow considerations in stroke centers, while acknowledging the limitations inherent to the observational study design.

Future prospective, multicenter, and ideally randomized studies are warranted to validate these findings, further characterize patient radiation exposure outcomes, assess the impact of dual operators on long-term clinical outcomes, and evaluate the cost-effectiveness of this approach.

## 5. Conclusions

Performing mechanical thrombectomy with two experienced operators in acute ischemic stroke was associated with shorter procedure times, lower patient radiation exposure, and higher angiographic success rates compared with single-operator procedures. These findings support an association between operator number and procedural performance metrics; however, given the retrospective design and organizational confounding, they should be interpreted as associative rather than causal. Further prospective, multicenter studies are warranted to clarify the independent impact of operator number on procedural efficiency, angiographic outcomes, and long-term clinical results.

## Figures and Tables

**Table 1:** Distribution of patients by occlusion level and operator count with baseline clinical characteristics.

Occlusion Level/Variable	All Patients (*n* = 285)	Single Operator (*n* = 157)	Dual Operators (*n* = 128)	*p*-Value
Demographic and Clinical Characteristics				
Age, years, median [IQR]	75 [62–82]	76 [62–82]	73 [63–81]	0.189
Sex, male (n, %)	147 (51.6%)	83 (52.9%)	64 (50.0%)	0.472
NIHSS, median [IQR]	12 [8–17]	12 [8–17]	13 [8–17]	0.460
ASPECT, median [IQR]	8 [7–9]	8 [7–9]	8 [7–9]	0.763
tPA administration (n, %)	61 (21.4%)	29 (18.5%)	30 (23.4%)	1.000
**Occlusion Site, *n* (%)**				
ICA terminal/Tandem	69 (24.2%)	44 (28.0%)	25 (19.5%)	
MCA M1–ACA A1	124 (43.5%)	61 (38.9%)	63 (49.2%)	
MCA M2–ACA A2	53 (18.6%)	32 (20.4%)	21 (16.4%)	
Basilar–PCA	39 (13.7%)	20 (12.7%)	19 (14.8%)	

Note: Statistical comparisons between single and dual operator groups were performed using the independent samples *t*-test for normally distributed continuous variables, the Mann–Whitney U test for non-normally distributed continuous variables, and Fisher’s exact test for categorical variables. Percentages in the “All patients” column are calculated over the total cohort (*n* = 285), while percentages in the “Single operator” and “Dual operators” columns are calculated over the respective group totals (*n* = 157 and *n* = 128). Abbreviations: NIHSS = National Institutes of Health Stroke Scale; ASPECT = Alberta Stroke Program Early CT Score; tPA = Tissue plasminogen activator; ICA = Internal Carotid Artery; MCA = Middle Cerebral Artery; ACA = Anterior Cerebral Artery; PCA = Posterior Cerebral Artery.

**Table 2:** Distribution of initial mechanical thrombectomy techniques between single- and dual-operator groups.

Technique	Single Operator *n* (%)	Dual Operator *n* (%)	Total *n* (%)	*p*-Value
Direct aspiration	59 (39.8)	55 (44.0)	114 (41.7)	
Stent retriever	25 (16.9)	17 (13.6)	42 (15.4)	
Solumbra	64 (43.2)	53 (42.4)	117 (42.9)	
*Total*	148 (100)	125 (100)	273 (100)	0.289

Note: Statistical comparison between single and dual operator groups was performed using the Chi-square test for categorical variables. Percentages in each column are calculated over the respective group totals.

**Table 3:** Comparison of procedural time and radiation dose parameters between single and dual operator groups.

Parameter	Single Operator (*n* = 157) Median [IQR]	Dual Operators (*n* = 128) Median [IQR]	*p*-Value
Procedure time (min)	85.0 [60.0–120.0]	52.5 [30.0–70.0]	<0.001
Fluoroscopy time (sec)	1627.0 [1040.0–2335.0]	1114.0 [718.5–2018.0]	<0.001
DSA run time (sec)	64.0 [41.0–103.0]	52.0 [36.0–78.2]	0.026
Total PKA (Gy·m^2^)	0.0092 [0.0060–0.0130]	0.0063 [0.0041–0.0100]	<0.001
Fluoroscopy PKA (Gy·m^2^)	0.0035 [0.0022–0.0052]	0.0023 [0.0015–0.0040]	<0.001
Imaging PKA (Gy·m^2^)	0.0050 [0.0031–0.0075]	0.0038 [0.0024–0.0061]	0.031

Note: Values are presented as median [interquartile range]. Statistical comparisons between single and dual operator groups were performed using the Mann–Whitney U test. Abbreviations: DSA = Digital Subtraction Angiography; PKA = Kerma-Area Product.

**Table 4:** Comparison of procedure time and radiation dose parameters by occlusion level and operator count.

Occlusion Level	Parameter	Single Operator, Median [IQR]	Dual Operators, Median [IQR]	*p*-Value
*ICA terminal/Tandem*	Procedure time (min)	90.0 [65.0–142.0]	60.0 [50.0–90.0]	0.0063
	Fluoroscopy time (sec)	1844.0 [1306.0–2524.0]	1895.0 [1050.0–2417.0]	0.3925
	Total PKA (Gy·m^2^)	0.0084 [0.0054–0.0119]	0.0081 [0.0065–0.0114]	0.9453
*MCA M1/ACA A1*	Procedure time (min)	75.0 [55.0–111.0]	40.0 [30.0–60.0]	<0.001
	Fluoroscopy time (sec)	1632 [1071–2266]	937.0 [646.0–1526.0]	0.0011
	Total PKA (Gy·m^2^)	0.0097 [0.0053–0.0135]	0.0058 [0.0034–0.0087]	<0.001
*MCA M2/ACA A2*	Procedure time (min)	62.0 [58.0–120.0]	60.0 [60.0–76.0]	0.1948
	Fluoroscopy time (sec)	1432 [898.0–3112]	1229.0 [846.0–1893.0]	0.6824
	Total PKA (Gy·m^2^)	0.0093 [0.0070–0.0124]	0.0058 [0.0043–0.0086]	0.0046
*Basiller/PCA*	Procedure time (min)	75.0 [55.0–120.0]	35.0 [30.0–60.0]	0.0016
	Fluoroscopy time (sec)	1685.0 [884.0–2404.0]	992.0 [692.0–1923.0]	0.1914
	Total PKA (Gy·m^2^)	0.0070 [0.0058–0.0158]	0.0071 [0.0049–0.0108]	0.2794

Note: Values are presented as median [interquartile range]. Statistical comparisons between single and dual operator groups for each occlusion level were performed using the Mann–Whitney U test. Abbreviations: PKA = Kerma-Area Product; IQR = Interquartile Range; ICA = Internal carotid artery; MCA = Middle cerebral artery; ACA = Anterior cerebral artery; PCA = Posterior cerebral artery.

**Table 5:** Reperfusion success rates (TICI ≥ 2b and TICI = 3) in patients with successful selective catheterization, stratified by operator count.

TICI Threshold	Single Operator (*n* = 157)	Success Rate (%)	Dual Operators (*n* = 128)	Success Rate (%)	*p*-Value
*TICI ≥ 2b*	101	64.3%	103	80.5%	0.004
*TICI = 3*	92	58.5%	98	76.6%	0.002
*Successful catheterization*	148	94.3%	125	%97.7	0.26

Note: Statistical comparisons between single- and dual-operator groups were performed using Fisher’s exact test. Abbreviations: TICI = Thrombolysis in Cerebral Infarction.

**Table 6:** TICI success rates (≥2b and 3.0) according to occlusion level and number of operators.

Occlusion Group	TICI Threshold	Single Operator (*n*, %)	Dual Operator (*n*, %)	*p*-Value
*ICA/ICA–MCA Tandem*	≥2b	34/44 (77.3%)	24/25 (96.0%)	0.0472
	=3.0	25/44 (56.8%)	22/25 (88.0%)	0.0080
*MCA M1/ACA A1*	≥2b	48/61 (78.7%)	57/63 (90.5%)	0.0836
	=3.0	37/61 (60.7%)	49/63 (77.8%)	0.0513
*MCA M2/ACA A2*	≥2b	25/32 (78.1%)	16/21 (76.2%)	1.0000
	=3.0	19/32 (59.4%)	13/21 (61.9%)	1.0000
*Basiller/PCA*	≥2b	16/20 (80.0%)	18/19 (94.7%)	0.3416
	=3.0	14/20 (70.0%)	18/19 (94.7%)	0.0915

Note: Statistical comparisons between single- and dual-operator groups were performed using Fisher’s exact test. Abbreviations: TICI = Thrombolysis in Cerebral Infarction; ICA = Internal Carotid Artery; MCA = Middle Cerebral Artery; ACA = Anterior Cerebral Artery; PCA = Posterior Cerebral Artery.

**Table 7:** First pass success, modified first pass effect, and mean number of passes after successful selective catheterization according to operator count.

Parameter	Single Operator (*n* = 148)	Dual Operator (*n* = 125)	*p*-Value
First Pass Success (FPS)	66 (44.5%)	75 (60.0%)	0.0146
Early reperfusion without rescue (ERR)	130 (88.4%)	115 (92.0%)	0.4164
Mean number of passes	2.00 ± 1.34	1.66 ± 1.02	0.0057

Note: *p*-values for FPS and ERR were calculated using Fisher’s exact test. The *p*-value for the mean number of passes was calculated using Student’s *t*-test. Abbreviations: FPS = First Pass Success; ERR = Early reperfusion without rescue; SD = Standard Deviation.

## Data Availability

The datasets generated and analyzed during the current study are available from the corresponding author upon reasonable request.
